# Water vapor transport properties of bio-based multilayer materials determined by original and complementary methods

**DOI:** 10.1038/s41598-023-50298-8

**Published:** 2024-01-02

**Authors:** Manon Guivier, Chloé Chevigny, Sandra Domenek, Joel Casalinho, Patrick Perré, Giana Almeida

**Affiliations:** 1 Université Paris-Saclay, INRAE, AgroParisTech, UMR SayFood, 91120 22 Place de l’Agronomie, Palaiseau, France; 2 CentraleSupélec, Laboratoire de Génie des Procédés et Matériaux, Université Paris-Saclay, 91190 Gif-Sur-Yvette, France; 3 CentraleSupélec, LGPM, Centre Européen de Biotechnologie et de Bioéconomie (CEBB), 3 Rue des Rouges Terres, 51110 Pomacle, France

**Keywords:** Materials science, Soft materials

## Abstract

To enhance PLA gas barrier properties, multilayer designs with highly polar barrier layers, such as nanocelluloses, have shown promising results. However, the properties of these polar layers change with humidity. As a result, we investigated water transport phenomena in PLA films coated with nanometric layers of chitosan and nanocelluloses, utilizing a combination of techniques including dynamic vapor sorption (DVS) and long-term water vapor adsorption–diffusion experiments (back-face measurements) to understand the influence of each layer on the behavior of multilayer films. Surprisingly, nanometric coatings impacted PLA water vapor transport. Chitosan/nanocelluloses layers, representing less than 1 wt.% of the multilayer film, increased the water vapor uptake of the film by 14.6%. The nanometric chitosan coating appeared to have localized effects on PLA structure. Moreover, nanocelluloses coatings displayed varying impacts on sample properties depending on their interactions (hydrogen, ionic bonds) with chitosan. The negatively charged CNF TEMPO coating formed a dense network that demonstrated higher resistance to water sorption and diffusion compared to CNF and CNC coatings. This work also highlights the limitations of conventional water vapor permeability measurements, especially when dealing with materials containing ultrathin nanocelluloses layers. It shows the necessity of considering the synergistic effects between layers to accurately evaluate the transport properties.

## Introduction

Bio-based and biodegradable materials are a potential substitute in packaging industry, which represents 41% of the total worldwide plastic production (according to *2021 Plastics Europe*), helping to decrease environmental pollution. Among the potential candidates, polysaccharides have attracted growing interest over the past decades because of their biodegradability, compostability and renewable origin^1–4^. Especially, nanocelluloses extracted from plant fibers have shown impressive mechanical and barrier properties and have recently been used in multiple industries such as biomedicine, papermaking, construction, textiles, packaging or electronics^5–10^. Among nanocelluloses, celluloses nanocrystals (CNC) and nanofibers (CNF) present interesting properties with different characteristics. CNC are nanometric rod-shape nanocelluloses with a high crystallinity (75–90%), while CNF are longer and flexible nanofibers (up to the micrometer^11^) with a lower crystallinity (up to 65%)^12,13^. Due to their dimensions, CNF have a high aspect ratio and form an entangled network^11^.

Moreover, the high number of free hydroxyl groups allows nanocelluloses to form a dense network highly barrier to gas molecules^14^, a property suitable for multiple applications, such as food packaging. Unfortunately, these free hydroxyl groups interact with water vapor in a high relative humidity environment, leading to nanocellulose swelling^15–18^. This phenomenon induces loss of barrier properties and have to be considered for food packaging applications.

To reduce their interaction with water, nanocelluloses and particularly CNC, can be combined with a hydrophobic polymer, such as polylactide (PLA)^16,19^, polypropylene (PP)^20^ or polyethylene (PE)^21^. Among them, PLA, a bio-based and biodegradable polymer with a water vapor permeability of 1–1.5 × 10^–14^ kg/m^2^ s Pa^22^, has received strong interest, particularly to obtain bio-based bulk composite reinforced with nanocelluloses. Because of poor compatibility between nanocelluloses and PLA, different types of surface compatibilization techniques have been developed: use of Beycostat surfactant^23^, CNC surface grafting with n-octadecyl-isocyanate^15^, PLLA(poly(l-lactide))-grafted with CNC^17^. Compatibilized nanocomposites showed an improvement in water vapor barrier properties, but compatibilization is costly. The oxygen barrier properties have been improved by a factor 3 to 8^16,17^ (down to 9.25 × 10^−19^ m^3^ m/m^2^ s Pa^16^). However, this improvement factors were not high enough to reach the properties of poly(ethylene terephthalate) (oxygen permeability of 1.1–5.3 × 10^−19^ m^3^ m/m^2^ s Pa^24,25^), a widely used petroleum-based polymer. Recently, we presented a novel 2D multilayer architecture employing thin nanocelluloses-coated layers on PLA, achieving impressive improvement factors in oxygen barrier properties ranging from 10 to nearly 100^26^. However, the interaction of these innovative materials with water molecules still needs to be investigated.

Water affinity of nanocelluloses has been studied mainly through evaluation of water vapor permeability of nanocelluloses films^27^: Tayeb et al*.*^28^ found a water vapor transmission rate (WVTR) of 400 g/m^2^ d (50–100% RH gradient), whereas Bedane et al*.*^29^ studied the WVTR at different relative humidity (from 10 to 100%) and obtained values from 29 to 792 g/m^2^ d. The large range of results obtained in literature does not provide explanations concerning the mechanisms of water absorption and transport in nanocelluloses, in particular concerning the impact of nanocelluloses swelling. However, bulk nanocelluloses films are characterized by high water mass uptake (up to 20%)^26^. Moreover, different nanocellulose types have distinct water absorption, which suggests that the specific characteristics of nanocelluloses play a crucial role in their interaction. In the same conditions, CNF TEMPO film absorbed approximately 50% more water than CNC or CNF films^26^. The effect of accelerated diffusion, resulting from swelling, on water vapor permeability requires further characterization and the distinct properties of nanocellulose materials should be considered in these assessments.

To explore these phenomena, PLA/nanocellulose multilayer films were fabricated. Building upon our previous research^26^, chitosan was selected as the tie layer due to its ability to form strong ionic interactions with nanocelluloses and a corona treatment was applied to PLA to enhance chitosan adhesion^30^. However, chitosan is also a highly hygroscopic polysaccharide and its impact on the water transport properties is critical for investigation. For instance, when a dry chitosan film is immersed in water, it can swell up to 400%^31^. Even at high relative humidity, it swells by over 100%^32,33^. Understanding the role of this layer in water transport properties is essential to comprehend the overall behavior of these multilayer films.

A simple, widely-used model to describe the overall permeability of multilayer film is the law of serial resistances (Eq. 1)^34^:

1 $$\frac{{L_{tot} }}{{P_{tot} }} = \mathop \sum \limits_{i = 1} \frac{{L_{i} }}{{P_{i} }},$$

where $L_{tot}$ and $L_{i}$ correspond to the thickness of multilayer film and each $i$ layer and $P_{tot}$ and $P_{i}$ to the permeability of multilayer film and of each layer $i$, respectively.

This law supposes an additive behavior of the layers and negligible interface effects between layers. Previous works have successfully used it to model the permeation of multilayer materials^34–36^. Garnier et al.^35^ described the permeation of water vapor through multilayers metallized PET materials with good accuracy between model and experiments.

In this study, the sorption and diffusion of water vapor in multilayer films were investigated using three complementary methods: water vapor permeation assessment (cup method), dynamic vapor sorption (DVS) and long-term permeation experiments where the water vapor passing through the film was measured with a relative humidity sensor at the downstream side of the film (back-face measurements)^37^. To gain deeper insights into the transport behavior of water molecules within the various nanocellulose layers, we employed an adaptation of a transport model developed by Perré et Turner (named *TransPoly*)^38^. Indeed, nanocelluloses layers can show anomalous diffusion, which implies a coupling between relaxation phenomena within the polymer matrix and mass transport. In the case of anomalous diffusion, there is an initial water vapor mass uptake dictated by Fick's law, followed by a continuous increase in water mass uptake that does not reach equilibrium within practical experimental timeframes. This non-Fickian mass uptake is attributed to the macromolecular relaxation of the polymer^39^. It has been well studied for PLA films^40,41^ and PLA/nanocellulose bulk composites^19,42^. *TransPoly* model incorporates coupled heat and mass transfer to fit the non-Fickian diffusion and provides with high precision the Fickian diffusion coefficient (D), the proportion of molecular relaxation (α) and the relaxation time (τ). *TransPoly* model was employed in conjunction with the DVS results and compared multilayer materials with individual thick films. This allowed us to better understand the water vapor transport properties in chitosan and nanocelluloses in either micrometric or nanometric layers. Analyses on various nanocellulose types were conducted (cellulose nanocrystals (CNC), cellulose nanofibrils (CNF), TEMPO-oxidized CNF (CNF TEMPO)) to understand how nanocellulose geometry and surface properties influence their behavior.

## Results

### Analysis of water vapor transport properties by gravimetric sorption experiments

**Figure 1:**
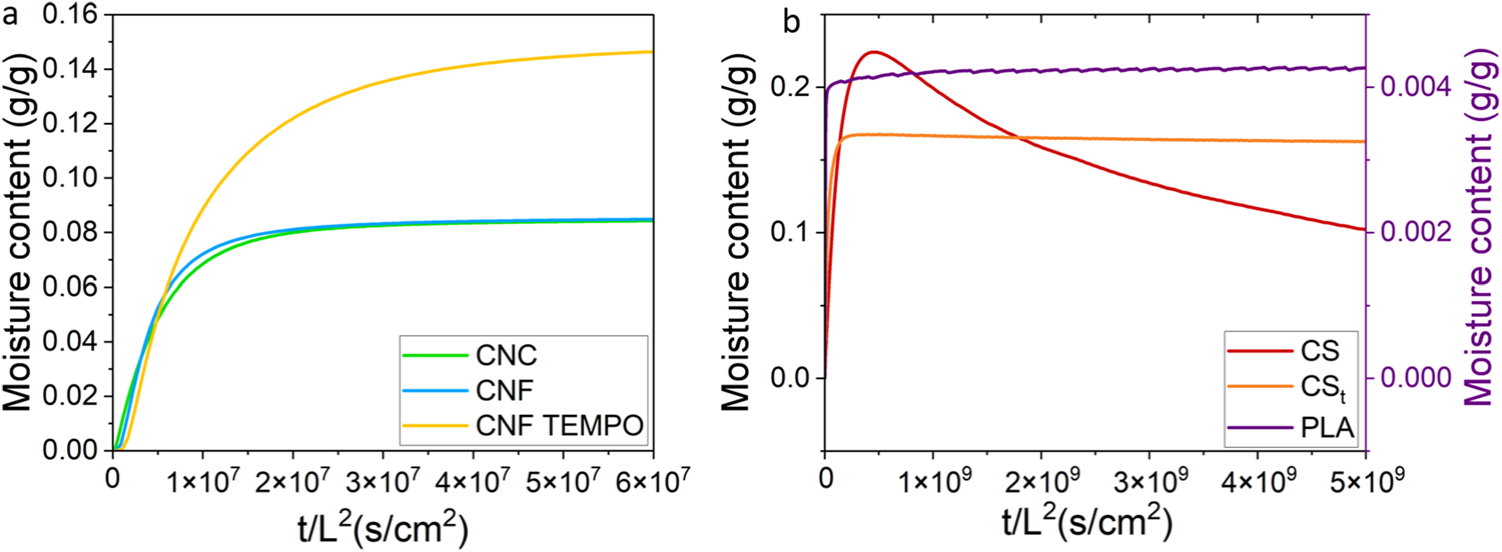
Moisture content of bulk nanocelluloses (**a**) and PLA, chitosan (CS) and treated chitosan (CS_t_) films according to the time normalized to the film thicknesses (0–80% RH step).

To understand the effect of each component of the multilayer film, sorption measurements of bulk films were realized. Figure 1 presents the moisture contents normalized to the thickness of bulk nanocelluloses and chitosan films. CNC and CNF films showed similar water vapor uptakes, whereas CNF TEMPO samples took up significantly more water with a moisture content 2 times higher than CNC and CNF films^26^. The chitosan film was treated by neutralization with NaOH solution, as recommended by He et al.^43^. Untreated (CS) and treated (CS_t_) chitosan films presented different behaviors (Fig. 1b). The untreated chitosan film exhibited a large overshoot, which disappeared after its neutralization. PLA moisture uptake is also represented in Fig. 1b. Due to its relatively high hydrophobicity, PLA took up 40 times less water than chitosan films.

The water absorption of the individual bulk samples, measured at 80% relative humidity (RH), is compiled in Table 1. Table 2 provides the mass percentage attributed to each layer, the experimental moisture content of each coated sample and the corresponding expected equilibrium moisture content. This equilibrium moisture content was calculated based on the bulk moisture content of each component, using Eq. 6. As expected, polysaccharide coatings increased the sample moisture content, although the coated layers were thin (below 500 nm), which represents a very low relative contribution in mass. The experimental moisture content is much higher that the value that would be expected by linear additivity using the sorption equilibrium of bulk materials (EMC, Table 2). For example, there was a large moisture content increase (12.8%) after the coating of the chitosan layer, although this layer was only 40 nm thick (less than 0.1% of PLA film thickness). Using the law of serial resistances (Eq. 4), each layer of multilayer architecture influences the sample permeability according to its thickness (or mass). In the case of PLA(CS) sample, PLA represented 99.9% of the sample mass and even though chitosan absorbed 40 times more than PLA, the calculated PLA(CS) water sorption should be only about 4% higher than the uncoated PLA film.

**Table 1:** Moisture content in bulk components of samples at 80% RH, with the corresponding film thickness.

Pure components	Thickness (µm)	Moisture content (MC) (g_water_/100 g _dry matter_)
PLA	50 ± 2	0.673
CS	70 ± 13	26.1
CNC	120 ± 11	11.9
CNF	95 ± 13	13.1
CNF TEMPO	85 ± 6	20.1

Experimental variability: 10%.

**Table 2:** Moisture content moisture content in PLA and coated samples at 80% RH, from measurements (MC) and from calculations (EMC), with the thickness and mass in percentage of PLA and the coatings.

	Thickness		Mass (%)		Moisture content (g_water_/100 g _dry matter_)	
Sample	PLA (µm)	CS/NC layer (nm)	PLA	CS/NC layer	Expected MC (sample)	MC (sample)
PLA (1)	50 ± 2	–	100	–	–	0.673
PLA (2)	50 ± 2	–	100	–	–	0.620
PLA(CS)	50 ± 2	40	99.98	0.02	0.691	0.759
PLA(CS)(CNC)_2_	50 ± 2	120	99.76	0.24	0.712	0.732
PLA(CS)(CNC)_10_	50 ± 2	440	99.13	0.87	0.797	0.759
PLA(CS)(CNF)_2_	50 ± 2	44	99.91	0.09	0.692	0.744
PLA(CS)(CNF)_10_	50 ± 2	60	99.88	0.12	0.697	0.763
PLA(CS)(CNF TEMPO)_2_ (1)	50 ± 2	48	99.90	0.10	0.695	0.747
PLA(CS)(CNF TEMPO)_2_ (2)	50 ± 2	48	99.90	0.10	0.695	0.771
PLA(CS)(CNF TEMPO)_10_ (1)	50 ± 2	80	99.84	0.16	0.710	0.697
PLA(CS)(CNF TEMPO)_10_ (2)	50 ± 2	80	99.84	0.16	0.710	0.716

Experimental variability: 10%

EMC(sample) : Expected equilibrium moisture content in the sample, MC(sample): Moisture content in the sample.

After the addition of nanocelluloses layers, the moisture content in multilayer materials increased due to the water uptake within nanocelluloses layers. Interestingly, the expected moisture content of PLA(CNC)_10_ was slightly higher than what was experimentally measured. This could be potentially attributed to the denser structure of coated CNC layers compared to bulk film, as well as the ionic bonding between chitosan and CNC. On the contrary, the expected moisture content of PLA(CNF)_10_ was smaller than the experimental value. CNF do not carry negative charges, in that case a denser structure caused by ionic bonds could not be obtained and coated CNF took up slightly more water molecules than bulk CNF film. The mass uptake of PLA(CNF TEMPO)_10_ coating was consistent with the expectation. The possibility to form strong interactions with chitosan did apparently not impact their water uptake capacity.

**Figure 2:**
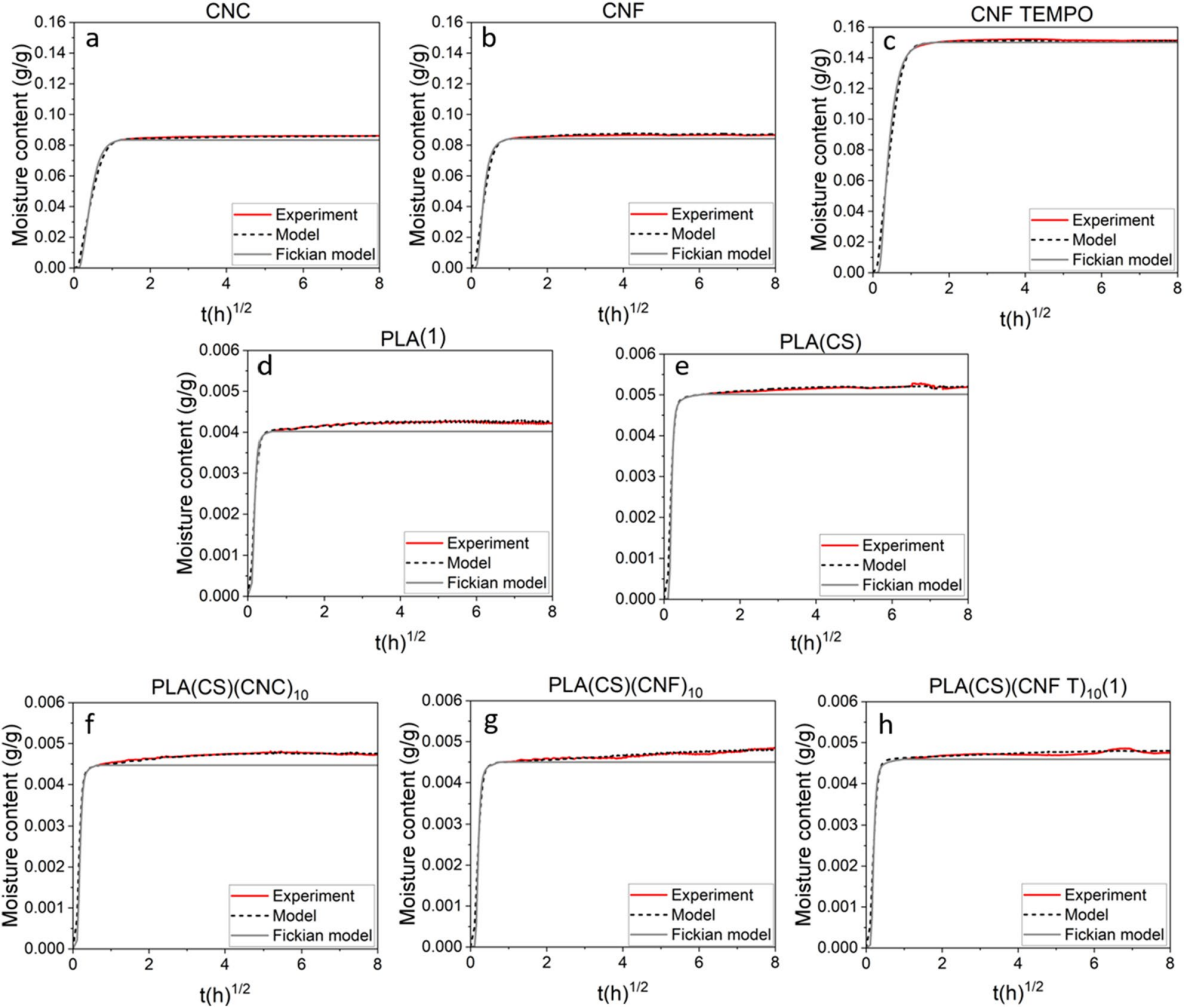
Water vapor sorption kinetics of 30–80% RH step, for bulk, uncoated and coated-PLA films with 10 layers of nanocelluloses. The experimental data are represented in red, *TransPoly* fit in black dashed line and the pure Fickian sorption behavior in grey.

The water vapor uptake kinetics were analyzed using *TransPoly* model. Figure 2 shows the quality of the fit and the resulting diffusion coefficient (D), molecular relaxation portion (α), and relaxation time (τ) are summarized in Table 3. The experimental conditions were used as boundary conditions for the analysis.

**Table 3:** Thickness of the bulk films, uncoated and coated-PLA samples, and mass transport parameters obtained by *TransPoly* model for the 30–80% RH step.

Samples	Film thickness (µm)	CS/NC layer thickness (nm)	D × 10^−12^ (m^2^/s)	α (%)	τ (h)
CNC (bulk)	120 ± 11	–	1.5	1.6	20
CNF (bulk)	95 ± 13	–	0.5	2.0	22
CNF TEMPO (bulk)	85 ± 6	–	0.4	0.8	4.1
PLA (1)(bulk)	50 ± 2	–	2.3	5.0	6.9
PLA (2) (bulk)	50 ± 2	–	1.8	6.1	14
PLA(CS)	50 ± 2	40	2.5	3.5	4.1
PLA(CS)(CNC)_2_	50 ± 2	120	2.2	5.5	2.7
PLA(CS)(CNC)_10_	50 ± 2	440	3.1	5.5	7.1
PLA(CS)(CNF)_2_	50 ± 2	44	2.0	4.0	1.8
PLA(CS)(CNF)_10_	50 ± 2	60	1.8	6.2	24
PLA(CS)(CNF TEMPO)_2_(1)	50 ± 2	48	2.4	6.1	8.5
PLA(CS)(CNF TEMPO)_2_(2)	50 ± 2	48	1.8	7.5	24
PLA(CS)(CNF TEMPO)_10_(1)	50 ± 2	80	1.9	5.2	8.6
PLA(CS)(CNF TEMPO)_10_(2)	50 ± 2	80	1.9	3.2	15

D: diffusion coefficient, α: relaxation proportion, τ: relaxation time.

The model accurately described the initial rapid moisture uptake following Fick’s law and the second slow uptake due to relaxation. Uncoated and coated-PLA films showed a Fickian water vapor uptake for approximately 30 min, whereas it lasted approximately 1h in bulk nanocelluloses films. This was correlated to the polysaccharide film thickness (between 85 and 120 µm). The slow second moisture uptake corresponded to a non-Fickian behavior^42^ and was similar for all types of coatings.

As observed in Fig. 2 and Table 3, the water vapor diffusion in bulk nanocelluloses films showed a relaxation proportion at least twice smaller than PLA. The diffusion coefficient of CNC was found to be 1.54 × 10^–12^ m^2^/s, similar to the result obtained by Belbekhouche et al.^44^ (1 × 10^–12^ m^2^/s, at 80% RH). According to Tayeb et al.^45^, the diffusion coefficient of water vapor in CNF film is approximately 2.4 × 10^–12^ m^2^/s (at 90% RH), which is 4 times higher than the value obtained. The difference may be attributed to the higher RH selected by the authors for their experiments. The diffusion coefficient in bulk nanocelluloses film varies with the relative humidity and moisture content.

The diffusion coefficient of the uncoated PLA film (2.3 × 10^–12^ m^2^/s) was consistent with literature values^40,46^. Considering the uncertainty of the measurement of the diffusion coefficient, we cannot clearly distinguish the slow-down caused by nanocellulose coatings.

The Fickian diffusion coefficient and the relaxation amplitude of multilayer materials were driven by the PLA bulk layer. Indeed, as shown in Table 3, bulk nanocelluloses films had a lower relaxation proportion than PLA. Thus, the deposition of nanometric nanocelluloses layers on PLA should not affect PLA relaxation.

### Water vapor permeability of uncoated and coated PLA (cup methods)

**Figure 3:**
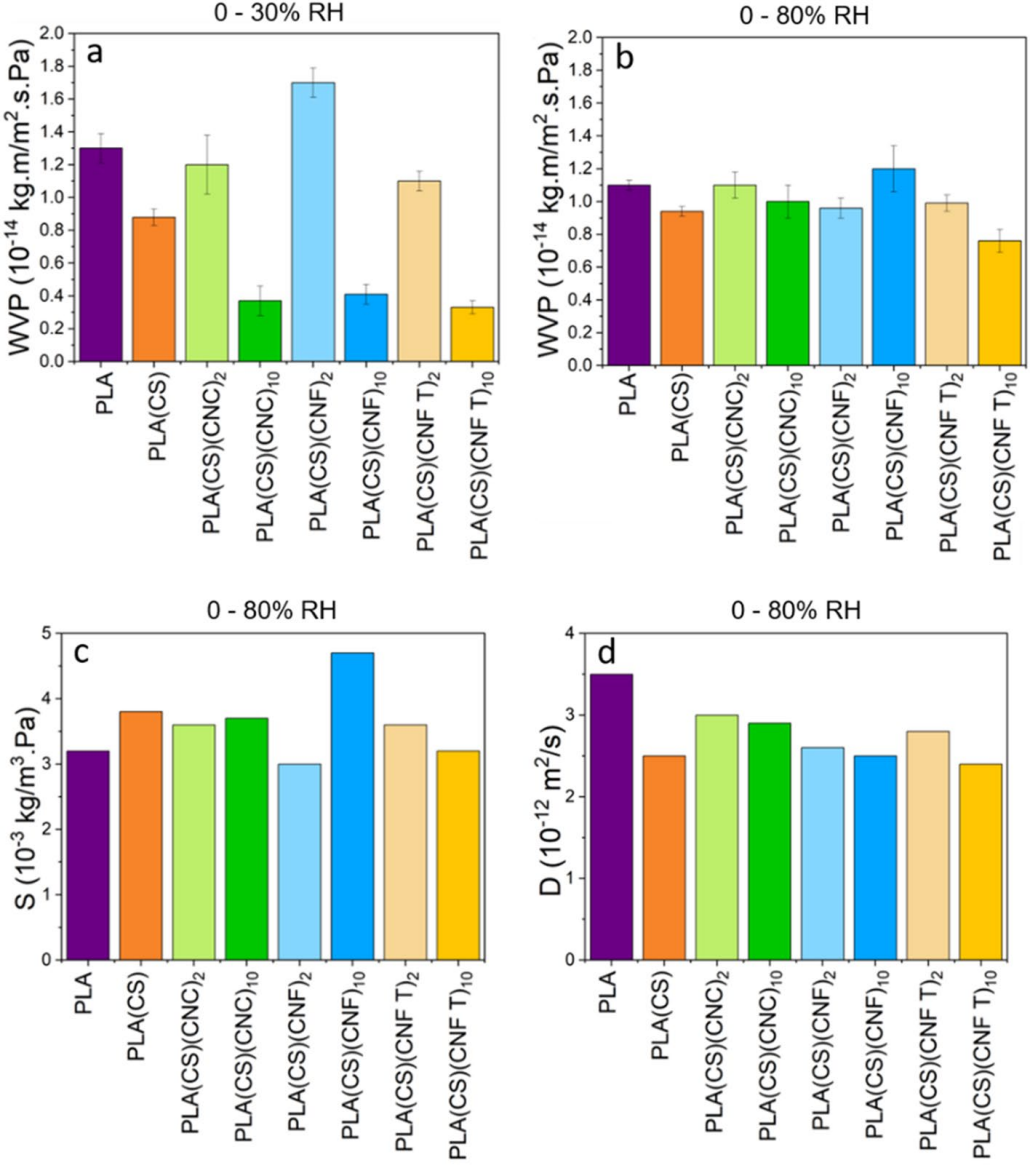
Transport coefficients of PLA, PLA(CS) and coated-PLA films calculated from cup and DVS methods. (**a**) WVP (water vapor permeability) at 0–30% RH calculated with cup methods. (**b**) WVP at 0–80% RH calculated with cup methods. (**c**) Solubility at 0–80% RH calculated with cup and DVS methods. (**d**) Diffusion at 0–80% RH calculated with cup methods.

The gravimetric mass uptake kinetics allow the independent determination of diffusion and solubility coefficients and can be used to calculate the sample permeability. Indeed, permeability is the macroscopic property of technological relevance. Figure 3 summarizes the WVP of samples for the 0–30% RH (3a) and 0–80% RH steps (3b) and the corresponding solubility (3c) and diffusion coefficients (3d) for the 0–80% RH step. The solubility coefficients were extracted from DVS results and used to compute the diffusion coefficient of coated samples. PLA WVP results were similar to literature (10^–16^–10^–13^ kg.m/m^2^.s.Pa)^47^. For the 0–30% RH step, a decrease in WVP is observed for samples coated with 10 layers of nanocelluloses (up to 76%), whereas 2 layers of nanocelluloses seem to not affect PLA WVP. Adding thicker layers of nanocelluloses improved the barrier properties of coated samples. However, when the relative humidity increased to 80%, the water completely disturbed nanocelluloses barrier properties. Because of nanocelluloses swelling, similar WVP values for uncoated and coated PLA at 80% RH were observed, except in the case of 10 layers of CNF TEMPO. CNF TEMPO presented the strongest interactions with chitosan. A potential explanation might be that these interactions prevented the coating swelling and maintain the barrier performance at higher RH.

Nanocelluloses coatings seems to increase the sample water solubility (Fig. 3c), as the coatings are very hydrophilic. The sample with 10 CNF layers had the highest solubility coefficient. CNF layer does not carry charges, therefore its interactions with chitosan are weaker. Diffusion coefficients in Fig. 3 and Table A (Supporting information) were calculated according to Eqs. (7) and (8), using the solubility and permeability values. Overall, the results agreed with the one obtained by DVS measurement and calculated from *TransPoly* fit. A slight decrease in diffusion coefficient with respect to PLA was observed.

### Water vapor transport properties determined by long-term permeation experiments

The water vapor diffusion through samples submitted to a relative humidity of 80% was studied over the long-term with back face measurements, i.e. measurement of the increase of relative humidity at the downstream side of a multilayer film. The advantage of this method, which waits for the equilibrium of RH in the up and downstream compartment, is that the mass transport parameters can be modeled with higher reliability because the equilibrium value is known. To our knowledge, a measurement of water vapor transport through multilayer architecture lasting 34 days (51 days considering the equilibrium steps) was not realized before, although it is highly interesting to study polymer relaxation.

**Figure 4:**
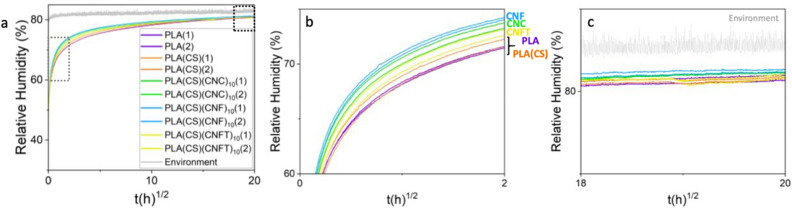
Evolution of relative humidity measured at the downstream side of samples during the back-face measurements (30–80% RH) (**a**) and the corresponding zooms (**b**,**c**) indicated by grey (**b**) and black (**c**) squares in Figure (**a**).

Figure 4 presents the experimental curves of the relative humidity increase inside the closed chamber for uncoated and coated PLA samples with chitosan and 10 deposits of nanocelluloses. The polysaccharide face was oriented to the downstream side. For each sample, two measurements were performed (labeled (1) and (2), respectively), showing very good repeatability.

Coated PLA samples show a faster RH increase on the downstream compartment than uncoated PLA and chitosan-coated PLA (Fig. 4b). Interestingly, none of the experiments reached the equilibrium value of 80% RH on the back-face. This shows that the transport slowed down extensively during the experiment.

## Discussion

In this work and for the first time, water vapor transport properties of a multilayer materials using nanocelluloses were studied in detail with complementary experiments. In particular, a long-term permeation experiment of 34 days was carried out.

The analysis of the bulk properties of the different nanocelluloses showed that the geometric shape and surface charge of polysaccharide structures played a key role for the mass transport (Tables 1 and 2). CNC and CNF with respectively low and negligible surface charges exhibited equal water sorption capacity. However, the rod-shaped CNC, which yields films with intrinsic porosity, allowed for faster diffusivity of water compared to CNF, which are long, rigid fibers forming entangled films. CNF TEMPO fibers have a similar fiber structure as CNF, with a smaller fiber diameter. They formed entangled films with equivalent diffusivity to CNF films but exhibited higher equilibrium water uptake. The difference is explained by the high negative surface charge density on CNF TEMPO, which weakens fiber interactions and the smaller fiber diameters providing more sorption sites^48^.

Chitosan, unlike nanocelluloses, is not a fiber but a polymer which is soluble in acid aqueous solutions, with acetic acid being the most commonly used solvent. Upon solvent evaporation, it forms dense semi-crystalline films. The acid conditions impart a positive surface charge to the –NH_3_^+^ functional groups. The properties of chitosan films in high relative humidity environment can be adjusted by altering the surface charge through neutralization with bases like NaOH. The water mass uptake in acid chitosan showed an initial large overshoot, followed by a constant decrease in moisture content (Fig. 1b). This decrease might be attributed to crystallization of the chitosan. Other studies have shown that neutralization reduces chitosan swelling by altering its crystalline conformation and surface chemistry^32,43,49,50^. The decrease in swelling results from the formation of new hydrogen bonds during neutralization process, as supported by FTIR analysis, indicates deprotonation of chitosan by the conversion of NH_3_^+^ groups to NH_2_^32^. Besides, NaOH neutralized the remaining acetic acid in chitosan film and this explains the stronger interactions between chitosan molecules and the weaker swelling^32^.

In literature, chitosan/nanocelluloses films have been investigated^31,33,51^: Li et al.^33^ added CNC in a chitosan matrix with concentrations from 5 to 30 wt.%. The addition of CNC reduced chitosan water vapor uptake, mainly due to the high crystallinity of CNC and the strong interactions between CNC and chitosan. Resende et al.^51^ investigated the water vapor permeability of chitosan films reinforced with CNF. The presence of CNF induced a tortuous path and reduced the water vapor permeability. Based on these findings, we opted not to neutralize the chitosan films to capitalize on the opportunity for ionic interactions with nanocelluloses.

**Figure 5:**
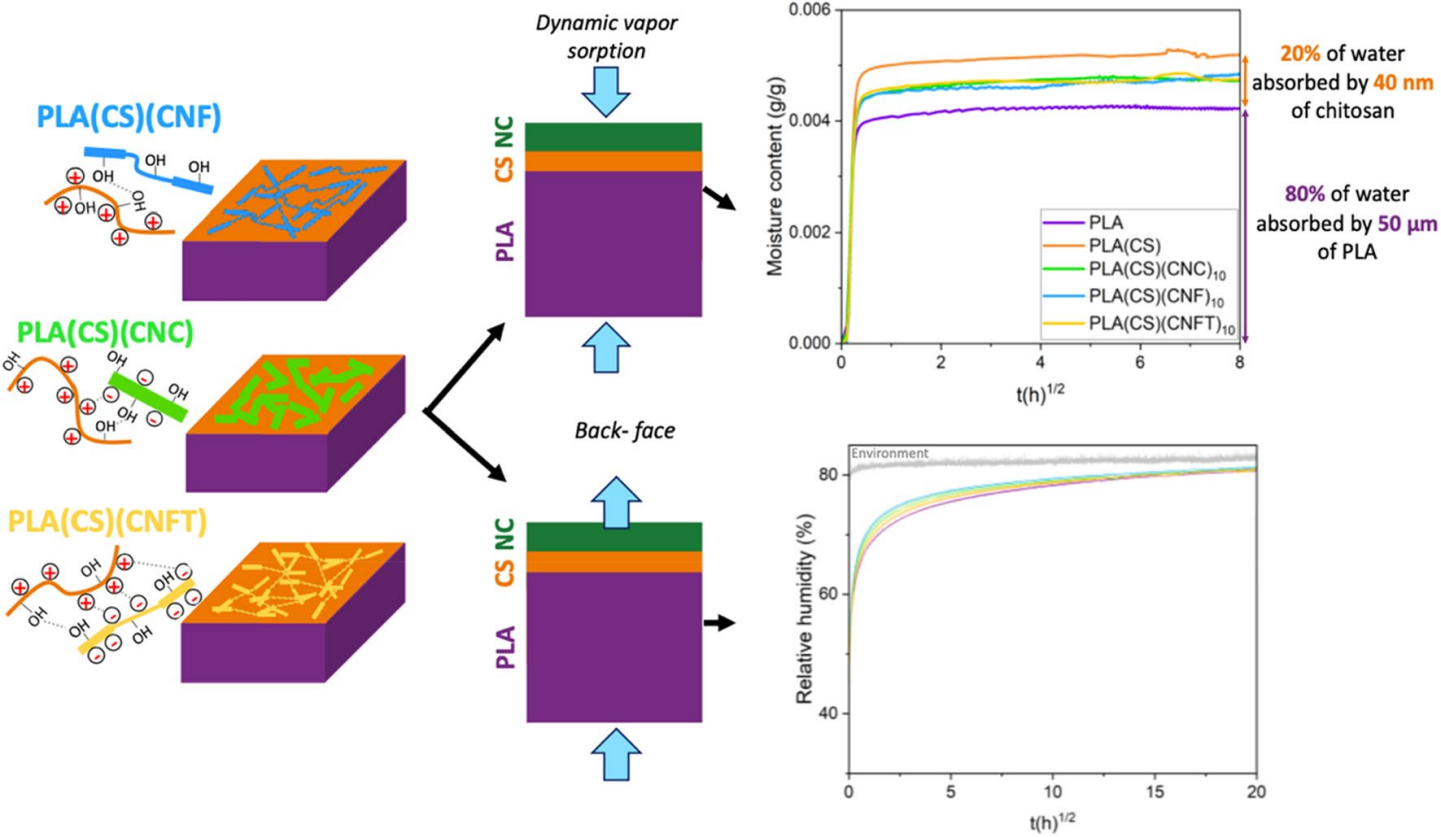
Summary of the DVS and back-face measurement principles and main results for multilayer films with CNC, CNF and CNF TEMPO coatings.

The analysis of the equilibrium water mass uptake of PLA(CS) and PLA(CS)(NC) multilayer samples showed the importance of chitosan/nanocellulose interactions, as illustrated in Fig. 5. The coating of a chitosan ultrathin layer (40 nm) (0.02% of chitosan in mass) increased the equilibrium moisture content by 12.8% (from 0.620 to 0.759 g/100 g, Table 1). Attributing the total mass uptake to the chitosan layer, this means that it took up more water than in the highest point of the overshoot in Fig. 1b. All supplementary nanocelluloses coatings reduced chitosan swelling (Fig. 5). The surface charge of nanocelluloses played an essential role in the swelling properties of polysaccharide layers. Effects were higher with 10 deposits. Neutral CNF coatings exhibited the highest mass uptake, surpassing the expected values calculated from the bulk properties of individual layers (Table 1). In contrast, the highly negatively charged CNF TEMPO showed the lowest mass uptake. The moderately charged CNC layers displayed intermediate mass uptake, which was less than expected (Table 1). This phenomenon can likely be attributed to both the structuring effect of chitosan on nanocellulose layers and the layer deposition method used for CNC (bar-coating), which led to a denser structure compared to solvent-casted films. In conclusion, the combination of chitosan and nanocelluloses is an efficient solution to improve the water resistance of coatings.

Diffusivity of water in multilayer films was analyzed using the mass uptake kinetics of DVS measurements and WVP curves obtained by the cup method. DVS kinetics revealed that multilayer materials displayed non-Fickian diffusion, characterized by an initial rapid mass uptake followed by a gradual and slower increase. Non-Fickian diffusion of water in PLA^41^ and PLA/nanocelluloses composites^42^ has been previously documented in literature. *TransPoly* model provided an accurate description of this behavior. Diffusion coefficients obtained from the cup method (Table 3) and *TransPoly* fit (Table 2) were similar, demonstrating consistency and high reliability of the two methods used to obtain these results. However, differences in the overall diffusion coefficient of the materials could not be reliably interpreted, because of the too small differences. Diffusion coefficients of nanocelluloses layers were not sufficiently different from PLA to show a barrier effect with films of a few tens or hundreds of nanometers. With the help of *TransPoly* fitting parameters, we observed the influence of nanometric coatings on PLA relaxation (Table 2). Bulk nanocelluloses films had a lower relaxation proportion than PLA, while coated samples had equal or slightly higher relaxation proportions than uncoated PLA. The relaxion time of coated samples with 10 layers tended to be higher than that of PLA. Therefore, it might be possible that coated layers interact with the PLA layer and slightly affect its structure, thus increasing its relaxation.

The WVP was analyzed with the cup method because of the technological relevance of this parameter. At low relative humidity, a reduction of the permeability was observed although nanocelluloses are hygroscopic materials. This effect increased with increasing nanocelluloses layers number. However, at high relative humidity, the barrier effect diminished due to polysaccharide swelling, except for the PLA(CS)(CNF TEMPO)_10_ sample. The extent of ionic interactions between chitosan and nanocellulose played a crucial role in this behavior, aligning with the insights gained from the analysis of fundamental transport parameters. In conclusion, the CS/CNF TEMPO combination shows potential to develop performing barrier coatings in an extended range of RH.

While the cup method is convenient, it has limitations as it cannot capture transient phenomena and diffusive behaviors like anomalous diffusion. Back-face measurements offer continuous, long-term monitoring in a more realistic scenario where concentrations reach equilibrium over time across the membrane. For modeling, this approach has the advantage of a known equilibrium state. Interestingly, in the extended analyses (after 17 days at 80% RH), back-face measurements did not reveal significant differences between uncoated and coated samples (Fig. 5). However, on short-time analysis, nanocelluloses coated samples diffused more rapidly than PLA (Fig. 4b). There is no physical reason for the addition of two resistances to decrease the overall barrier properties of the complex. We hypothesize that the faster diffusion of water molecules in the presence of coating can be explained by the plasticizing of PLA with remaining acetic acid from chitosan. Indeed, Chang et al.^32^ showed that chitosan neutralization removed the remaining acetic acid in chitosan films. Plasticizers bring additional free volume to a polymer and are generally negatively impacting barrier properties^52^. We present in the supporting information (Table B) data showing the decrease of the glass transition temperature of coated PLA (from 54.4 ± 0.4 to 53.4 ± 0.5 °C). The plasticizing of PLA at its interface could also be an additional explanation for the high water uptake of PLA(CS) films (Table 1), which could add up with the water uptake of the chitosan layer. The highest acceleration of the transport was reached with CNF coatings which have the weakest interactions with chitosan. However, the sample with only chitosan coating (PLA(CS)) did not present the faster diffusion as expected and further investigations are needed to validate the hypothesis of PLA plasticization with acetic acid.

Interestingly, all samples reached a similar relative humidity after 34 days, although they did not achieve equilibrium RH due to the very slow diffusion of water molecules through PLA membrane at high RH (Fig. 5). This suggests that the diffusivity in the membrane decreased over time. A possible explanation for this behavior is the formation of water clusters at high RH^41,53,54^. Water clusters increase the volume of the transported unit, which slows down the transport rate. While a decrease in diffusivity of water in PLA at high RH has been previously described^40,41^, experimental evidence for this phenomenon still remains limited. These findings show that the back-face measurements are a very sensitive and accurate tool for the investigation of complex mass transport phenomena.

## Conclusion

The combination of water vapor permeability (WVP) measurements, dynamic vapor sorption (DVS), and back-face measurements investigated the water vapor transport properties of PLA samples coated with a chitosan tie layer and different types of nanocelluloses. This challenging characterization clarified the interactions between these nanometric coatings.

The findings showed the significant importance of synergy between layers. Chitosan layer had a noticeable impact on the local free volume and water vapor transport properties of PLA film. This was most probably caused by the presence of plasticizing molecules, such as acetic acid. The synergy between chitosan and nanocelluloses layers played a key role in the sample water barrier properties. The establishment of strong ionic interactions between positively charged chitosan and negatively charged nanocelluloses significantly reduced their water affinity. Consequently, CNF TEMPO layers, with the highest density of negative surface charges, exhibited the greatest resistance to water vapor transmission, whereas CNF, without negative surface charges, displayed the lowest resistance.

This original work contributes to a better understanding of multilayer architecture and facilitates the distinction of nanocellulose types based on their water affinity. The results offer valuable insights for selecting the right architecture and materials to construct new nanolayered samples using nanocelluloses.

## Experimental section

### Materials

PLA biaxially oriented film (density: 1.25 g.cm^-3^, thickness: 50 µm) was purchased from Goodfellow (UK). Acetic acid (99%-100%) was purchased from Sigma-Aldrich (USA). Medium molecular weight chitosan (molecular weight range: (1.9–3.1) × 10^5^ Da) from deacetylated chitin (75–85% deacetylation degree) was purchased from Sigma-Aldrich (USA). The deacetylation degree and molecular weight of chitosan were conformed to literature: 76%^55^ and 78%^56^ and 361 + /- 32 kDa^55^ or 583 + /- 87 kDa^56^. Cellulose nanocrystals (CNC), cellulose nanofibers (CNF), and cellulose nanofibers TEMPO-oxidized (CNF TEMPO) suspensions were purchased from the University of Maine (USA), with respective concentrations of 10.3 wt.%, 3 wt.% and 1.1 wt.%. Their characterization was performed by Jimenez-Saelices et al.^48^. The average dimensions of CNC were 6.5 × 20 × 175 nm^3^, they had a negative surface charge (OSO_3_^-^) of 0.090 ± 0.024 mmol/g and a crystallinity degree of 88.9–92.3%. CNF have an average diameter of 40–120 nm and a length up to several micrometers, with no detectable surface charge and a crystallinity degree of 69.3%^12^. The average diameter of CNF TEMPO was between 3 and 8 nm, with a length of several micrometers, and a negative surface charge (COO^−^) of 1.084 ± 0.04 mmol/g. These values are confirmed by other works^57,58^.

### Sample preparation

#### Solution preparation

An aqueous solution of chitosan with acetic acid (respective concentration of 1 wt.%) was prepared and maintained under stirring (500 rpm, 4 h), followed by an ultrasound treatment (20% amplitude, 5 min) (Ultrasonic processor, Bioblock Scientific, France). Nanocelluloses (NC) solutions were prepared at optimized concentrations of 1 wt.%, 0.1 wt.% and 0.05 wt.% for CNC, CNF TEMPO and CNF, respectively. All solutions were kept under agitation (500 rpm, 2h) before an ultrasound treatment (30% amplitude, 5 min).

#### Film preparation

Multilayer films and bulk chitosan and nanocelluloses films were prepared with bar-coating and solvent casting.

### Multilayer films

A mini Corona from Boussey-Control (frequency: 5 MHz, voltage: 48 kV and length electrode: 6.5 cm) was applied on PLA surface to enhance the chitosan layer adhesion. The optimal parameters of the corona treatment were determined in our earlier work^26^. Then, PLA films were bar-coated with an adhesive layer of chitosan and multiple nanocelluloses layers. Between each coating step, samples were kept at 40 °C for at least 15 min to dry. Two films per nanocellulose type were prepared, with 2 and 10 deposits of nanocelluloses. Samples are labeled according to the baseboard, adhesive layer, nanocellulose type and the number of deposits, such as *PLA(CS)(nanocellulose type)*_*x*_, where “PLA” is the baseboard, “CS” is the adhesive layer of chitosan, “nanocellulose type” refers to either CNC, CNF or CNF TEMPO and “x” refers to the number of nanocellulose deposits (2 or 10). After the final coating, PLA(NC) films were deeply dried at 40 °C under vacuum (1.3 kPa) for 48 h, then stored in a desiccator over silica gel. Thickness of chitosan/nanocelluloses coatings were determined using the height scratching protocol adapted from Cranston and Gray^59^, detailed in our earlier work^26^. Sample cross-sections were also previously investigated by scanning electron microscopy and demonstrated nanocelluloses ultrathin coatings^26^.

### Bulk films

Chitosan, CNC, CNF and CNF TEMPO bulk films were prepared for dynamic vapor sorption measurements by solvent casting and stored in a desiccator prior to each measurement. Two chitosan films were prepared, with and without a neutralization process, respectively named CS_t_ and CS. The treated chitosan film (CS_t_), after the drying step was neutralized according to the protocol developed by He et al.^43^ The film was immersed in a NaOH solution ( concentration 10 wt.%) for 50 s, before being dried and stored in a desiccator. Thickness of bulk chitosan and nanocelluloses were measured with a Minigauge (ElektroPhysik, Germany).

### Characterization methods

#### Water vapor permeability

The sample water vapor transmission rate (WVTR) was measured at 25 °C and 80% RH. The procedure consists of measuring the weight uptake of dried CaCl_2_. The CaCl_2_ was placed in a glass cup, sealed with the sample film and tightened by the application of beeswax. The specific exchange surface was 15.2 cm^2^. To control the environmental conditions, the cups were placed in a desiccator with a relative humidity of 80.99 ± 0.28%, maintained with a salt solution ((NH_4_)_2_ SO_4_). The WVTR (kg.m/m^2^.s^1^) was obtained from the slope of the weight uptake, as presented in Eq. (2):

2 $$WVTR = \frac{slope \times l}{S},$$

where *slope* corresponds to the cumulated weight uptake against time (kg/s), $l$ to the sample thickness (m) and $S$ to the exchange surface (m^2^).

The WVTR was normalized to the water partial pressure to obtain the water vapor permeability, which considers the pressure gradient over the film, with Eq. (3):

3 $$WVP = \frac{WVTR}{{\Delta P}},$$

where $\Delta P$ is the gradient in partial pressure of water (Pa).

The normalized water vapor permeability of chitosan/nanocellulose (CS/NC) layer was calculated, according to Eq. (4):

4 $$\frac{{L\left( {sample} \right)}}{{WVP\left( {sample} \right)}} = \frac{{L\left( {CS/NC} \right)}}{{WVP\left( {CS/NC} \right)}} + \frac{{L\left( {PLA} \right)}}{{WVP\left( {PLA} \right)}},$$

where *L(sample), L(CS/NC)* and *L(PLA)* are the sample, CS/NC and PLA layer thicknesses, respectively. *WVP(sample), WVP(CS/NC)* and *WVP(PLA)* are their respective water vapor permeation values.

#### Dynamics vapor sorption (DVS)

Dynamic vapor sorption (DVS) from Surface Measurement Systems (UK) was used to determine the water sorption behavior of multilayered films, with a relative precision of 0.1 µg (± 1%). A specific pan was designed to optimize the contact surface between the sample and the water vapor, thus all the faces of the sample are exposed to the environmental condition. Measurements were performed under a continuous flow of nitrogen (270 cm^3^/min), at 25 °C. A three steps program was selected, with a first step at 0% relative humidity (RH) for 1440 min (1 d), to reach equilibrium and determine the anhydrous film mass. Then, a second step at 30% RH for 2880 min (2 d) and a third step at 80% RH for 4320 min (3 d) were realized. The duration of each step was optimized by *Espino-Pérez *et al.^19^, as a means to reach an equilibrium after each step ($dm/dt$ lower than 0.001%/min). Samples with a mass between 25 and 30 mg were characterized. Some selected samples were replicated to check repeatability and experimental error. All repetitions had similar results (standard deviation inferior to 2%), except for samples with CNF TEMPO coatings, for this reason, two repetitions are presented in the results.

The moisture content on a dry basis (MC) was calculated for each sample (corresponding to the water adsorbed), according to Eq. (5):

5 $$MC\left( {sample} \right) = \frac{{m_{humid} - m_{dry} }}{{m_{dry} }} \times 100,$$

where $MC\left( {sample} \right)$ is the moisture content of the sample (%), $m_{humid}$ the sample mass in a humid environment after reaching an equilibrium (g) and $m_{dry}$ the mass at the end of the first step, 0% RH (g).

The expected equilibrium moisture content (EMC) of the samples can be evaluated assuming a linear additivity of the layer contributions. With this assumption, the EMC Is simply an average, weighed by the respective mass of the moisture content of the pure components. The EMC was calculated according to Eq. (6):

6 $$EMC\left( {sample} \right) = \frac{{m\left( {PLA} \right)*MC\left( {PLA} \right) + m\left( {CS} \right)*MC\left( {CS} \right) + m\left( {NC} \right)*MC\left( {NC} \right)}}{{m\left( {sample} \right)}},$$

where $MC\left( {PLA} \right)$, $MC\left( {CS} \right)$ , $MC\left( {NC} \right)$ are the moisture content of pure PLA, bulk chitosan film and bulk nanocelluloses films (g_water_/100g_dry basis_) and $m\left( {PLA} \right)$, $m\left( {CS} \right)$, $m\left( {NC} \right)$, the respective mass PLA, chitosan and nanocelluloses layers and $m\left( {sample} \right)$ the sample mass (g).

The solubility coefficient S (kg/m^3^.Pa) was determined from the DVS measurements, according to the water concentration in the film after the relative humidity increase and the partial vapor pressure, as described in Eq. (7):

7 $$S = \frac{c}{p},$$

where *c* (kg/m^3^) is the concentration of water in the sample and *p* (Pa) is the partial vapor pressure.

Using the solubility coefficient determined with Eq. (7) and the WVP (or P) obtained with Eq. (3), the diffusion coefficient D (m^2^/s) can be calculated from Eq. (8):

8 $$D = \frac{WVP}{S},$$

where $WVP$ corresponds to the water vapor permeability, introduced in Eq. (3).

#### Back face measurements

**Figure 6:**
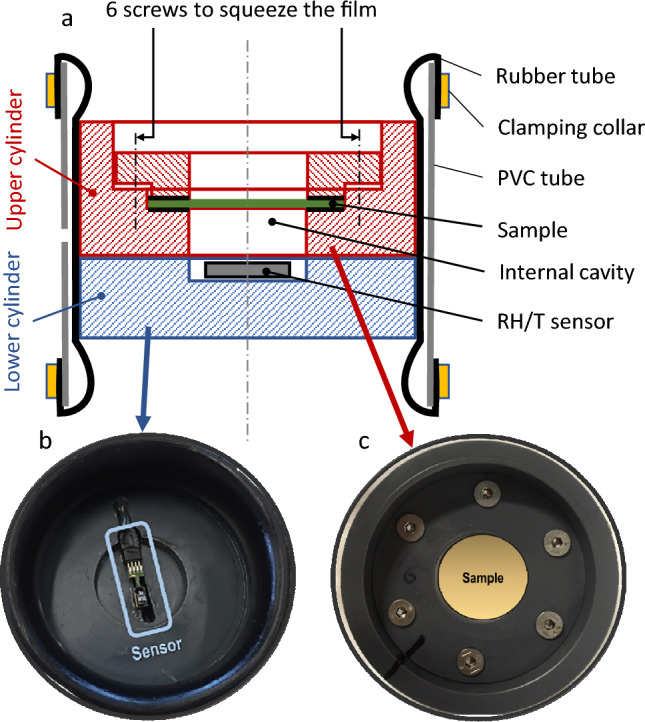
Schematic in-house back face set-up (**a**) with the corresponding top view of RH/T sensor holder (**b**) and PVC sample holder (**c**).

An in-house device, developed and described by *Perré *et al*.*^37^, was used to study the water vapor transfer through our samples for a long time (50 d). Figure 6 presents the in-house sample holder (5b, 5c) and its schematic cross-Sect. (5a). The coated PLA films were placed between two PVC holders and fixed in a PVC cylinder with a rubber liner and vacuum grease to avoid leaks. A vacuum pump is used to press the rubber liner against the PVC cylinder, allowing the placement of the sample holder inside the PVC cylinder. Then, the vacuum was stopped and the rubber liner perfectly fitted the PVC cylinder^60^. Each sample holder was put in a climatic chamber (Memmert, HPP110 Peltier technology for exterior climatic regulation; RH from 10 to 90 ± 0.5%; temperature from 0 to 70 ± 0.1 °C). The upper side of the sample holder withstands same temperature and relative humidity from climatic chamber regulation, whereas the bottom side contains an RH sensor (Sensirion SHT75; RH from 0 to 100 ± 1.8%; temperature from − 40 to 123.8 ± 0.3 °C) in a sealed environment. The experiment was performed during a 30%-80% RH step, until the equilibrium, fixed at $\frac{dRH}{{dt}} < 0.0014\% /h$, was reached. PLA(CS)(CNC)_10_, PLA(CS)(CNF)_10_ and PLA(CS)(CNF TEMPO)_10_ were analyzed in duplicate.

Prior to measurements, the RH sensors needed to be calibrated. SHT sensors are capacitive sensors with complementary metal oxide semiconductor (CMOS) technology that guarantees low noise and high-resolution readings. However, a sensor drift can be expected for long-time measurements due to prolonged exposure to high humidity. *Jankovec *et al*.*^61^ showed that after a long-time exposure under high humidity environments (higher than 80% RH), additional positive offset drift should be considered to the announced 3%. A reconditioning procedure needed to be performed to bring the sensor to its original calibration.

In this work, high relative humidity accuracy is required due to the low diffusion kinetics of water vapor through our samples. Therefore, a calibration protocol was developed to compensate the sensor offset at a given relative humidity and sensor drift over time. For the offset correction, the manufacturer reconditioning protocol was performed prior to each experiment. Six sensors were placed in six sample holders and two other sensors were positioned in the climatic chamber. The readings at the beginning give the initial RH offset (offset of each sensor to the average of all used sensors at 30% RH, without sample and right after the reconditioning procedure). Then, all coated PLA samples were placed in their sample holders and put in their initial position in the climatic chamber. The back-face experiment was started immediately.

At the end of the experiment, the samples were removed from their sample holders and new readings were performed to determine the final RH offset for each sensor. The corresponding offset of each sensor was obtained with linear regression. The two SHT inside the climatic chamber were used to estimate the sensor drift over a long-time measurement, considering the climatic chamber drift negligible. Prior to this work, we studied the climatic chamber drift using several probes. One of them was a high performance optical dew-point transmitter. These experiments allowed us to rule out climatic chamber drift.

#### Transpoly model for the analysis of sorption and permeation data

The DVS experimental data were analyzed with the inverse method developed by *Perré & Turner*^38^. A coupled heat and mass transfer model for solids is used with a 2-variables model, representing the diffusion in a one-phase product. This coupling, which tends to slow down the external transfer, must be accounted in the identification procedure to be accurate. *TransPoly* (Transfer through Polymer) was already applied to PLA/nanocelluloses bulk nanocomposites^19,42^. The model is based on the following assumptions:

- single phase containing the polymer $\rho_{s}$ and bound water $\rho_{b}$,
- the solid density is assumed to be constant $\rho_{s} = constant$,
- the water molecules migrate as bound water diffusion,
- the water enthalpy ($$h_{b} )$$ is assumed to be equal to the enthalpy of liquid water $$\left( {h_{l} } \right)$$ minus the differential heat of sorption, a decreasing function with increasing moisture content.

The simplified transport equations are presented as follows:

Moisture conservation

9 $$\frac{\partial X}{{\partial t}} = \nabla \left( {D_{b} \nabla X_{eq} } \right),$$

where $X$ and $X_{eq}$ are the moisture contents, dry basis and at equilibrium, respectively and $D_{b}$ is the diffusion coefficient.

Energy conservation

10 $$\frac{\partial }{\partial t}\left( {\rho_{s} \overline{{h_{b} }} + \rho_{s} h_{s} } \right) = \nabla \left( {{\uplambda }\nabla {\text{T}} + \rho_{b} h_{s} D_{b} \nabla X_{eq} } \right),$$

where $\overline{{h_{b} }}$ is the average enthalpy of bound water, $h_{s}$ is the specific enthalpy of the polymer, ${\uplambda }$ is the thermal conductivity, ${\text{T}}$ is the temperature and $\rho_{s}$ and $\rho_{b}$ are the density of the solid phase and bound water, respectively.

Boundary conditions

11 $$\left. {J_{v} } \right|_{{x = 0^{ - } }} n = h_{m} cM_{v} \ln \left( {\frac{{1 - x_{v,\infty } }}{{1 - x_{v,surf} }}} \right),$$

12 $$\left. {J_{q} } \right|_{{x = 0^{ - } }} n = h_{h} \left( {T_{surf} - T_{\infty } } \right),$$

where $J_{v}$ and $J_{q}$ correspond to the water vapor and the heat fluxes in the boundary, respectively. $x$ represents the molar fraction of water vapor, $T_{surf}$ the temperature at the exchange surface (x = 0) and $T_{\infty }$ the temperature outside of the boundary layer. $h_{h}$ and $h_{m}$ are the heat and mass transfer coefficients, *c* and the indices $\nu$ and $\infty$ stand for the conditions outside the boundary layer. *n* is the external unit normal.

Also, the difference between predicted and experimental values was quantified by an objective function S presented in the following equation:

13 $$S = \mathop \sum \limits_{i = 1}^{NT} w_{i} \left[ {M_{{{\text{exp}}}} \left( {t_{i} } \right) - M_{{{\text{pred}}}} \left( {t_{i} } \right)} \right]^{2} ,$$

where $M_{{{\text{exp}}}}$ and $M_{{{\text{pred}}}}$ are the experimental and predicted values, $w_{i}$ is the weighting coefficient, $t$ the time, and $NT$ is the number of time intervals.

In this work, we used a uniform weighting factor throughout the test: $w_{i}$, proportional to the time interval between two successive data acquisitions. An optimization algorithm (Simplex) was used to minimize this function by modifying the unknown parameters^62^. The diffusivity was given as the value reached after convergence. The pseudo-Fickian plateau value was determined as the average of the fitted plateau of the pseudo-Fickian curve.

The theoretical and normalized diffusion coefficients ($D_{theo}$) were also calculated with Eq. (14):

14 $$D_{theo} \left( {sample} \right) = \frac{{L\left( {PLA} \right) \times D\left( {PLA} \right) + L\left( {CS} \right) \times D\left( {CS} \right) + L\left( {NC} \right) \times D\left( {NC} \right)}}{{L\left( {sample} \right)}},$$

where *L(sample), L(CS), L(NC)* and *L(PLA)* are the sample and CS, NC and PLA layer thicknesses, respectively. *D(CS), D(NC)* and *D(PLA)* are the diffusion coefficients of bulk chitosan, bulk nanocelluloses and PLA films.

### Supplementary Information

Supplementary Tables.

## Data Availability

The datasets generated during and/or analyzed during the current study are available from the corresponding author on reasonable request.
