# Supplementary material for: Water vapor transport properties of bio-based multilayer materials determined by original and complementary methods
Source: Sci Rep. 2024 Jan 2;14:50. doi: 10.1038/s41598-023-50298-8 (PMC10761724; doi:10.1038/s41598-023-50298-8)
Supplement: Supplementary file 1 — Supplementary Tables. [file 41598_2023_50298_MOESM1_ESM.docx]

**Supporting information**

Table A: Transport coefficients at 0-30% RH and 0-80% RH steps, calculated with cup methods and DVS measurements.

| Sample | WVP (10^-14^ kg.m/m^2^.s.Pa)  0 - 30% RH | WVP (10^-14^ kg.m/m^2^.s.Pa)  0 - 80% RH | S (10^-3^ kg/m^3^.Pa)  0-80% RH | D (10^-12^ m^2^/s)  0-80% RH |
| --- | --- | --- | --- | --- |
| PLA | 1.3 ± 0.09 | 1.1 ± 0.03 | 3-3.2 | 3.5-3.7 |
| PLA(CS) | 0.88 ± 0.05 | 0.94 ± 0.03 | 3.8 | 2.5 |
| PLA(CS)(CNC)_2_ | 1.2 ± 0.18 | 1.1 ± 0.08 | 3.6 | 3.0 |
| PLA(CS)(CNC)_10_ | 0.37 ± 0.09 | 1.0 ± 0.10 | 3.7 | 2.9 |
| PLA(CS)(CNF)_2_ | 1.7 ± 0.09 | 0.96 ± 0.06 | 3.0 | 2.6 |
| PLA(CS)(CNF)_10_ | 0.41 ± 0.06 | 1.2 ± 0.14 | 4.7 | 2.5 |
| PLA(CS)(CNF TEMPO)_2_ | 1.1 ± 0.06 | 0.99 ± 0.05 | 3.6 | 2.7-2.8 |
| PLA(CS)(CNF TEMPO)_10_ | 0.33 ± 0.04 | 0.76 ± 0.07 | 3.2-3.8 | 2-2.4 |

Table B : Glass transition temperature (T_g_), melting temperatures (T_m1_, T_m2_) and crystallinity (X_c_) of PLA before and after its pre-treatment (PLA_treated_) and for PLA(CS)(CNC)10 sample.

| Sample | T_g_(°C) | T_m1_(°C) | T_m2_(°C) | X_c_(%) |
| --- | --- | --- | --- | --- |
| PLA | 54.4 ± 0.4 | 156 ± 0.6 | 167 ± 1 | 39.2 ± 1.8 |
| PLA_treated_ | 54.6 ± 0.2 | 157 ± 0.7 | 167 ± 1.4 | 40.6 ± 1.2 |
| PLA(CS)(CNC)_10_ | 53.4 ± 0.5 | 154 ± 1.7 | 168 ± 0.9 | 40.1 ± 3.2 |
